# Factors influencing concurrent wasting, stunting, and underweight among children under five who suffered from severe acute malnutrition in low- and middle-income countries: a systematic review

**DOI:** 10.3389/fnut.2024.1452963

**Published:** 2024-12-06

**Authors:** Godana Arero Dassie, Tesfaye Chala Fantaye, Tesfaye Getachew Charkos, Midhakso Sento Erba, Fufa Balcha Tolosa

**Affiliations:** School of Public Health, Adama Hospital Medical College, Adama, Oromia, Ethiopia

**Keywords:** concurrent wasting and stunting, underweight, severe acute malnutrition, under five, food insecurity

## Abstract

**Background:**

Wasting, stunting, and underweight in children are complex health challenges shaped by a combination of immediate, underlying, and systemic factors. Even though copious data demonstrates that the causation routes for stunting and wasting are similar, little is known about the correlations between the diseases in low- and middle-income nations.

**Objective:**

The objective of this study is to evaluate the factors that concurrently affect wasting, stunting, and underweight in <5-year-olds with severe acute malnutrition (SAM).

**Method:**

This review adhered to the Preferred Reporting Items for Systematic Reviews and Meta-Analyses (PRISMA) guidelines. We searched every electronic database that was available, from the medRxiv pre-print site, PubMed, MEDLINE, EMBASE, Cochrane Library, Web of Science, PsycINFO, CINAHL, Google Scholar, and Scopus, in addition to the Science Direct search engine. We considered research conducted in low- and middle-income nations on <5-year-olds with SAM. The Newcastle Ottawa Scale was used to assess the quality of the studies.

**Results:**

After screening and selecting 12 eligible studies, 1,434,207 records were included for analysis. The prevalence of factors influencing concurrent wasting, stunting, and being underweight was 26.42% in low-middle -income countries (LMI). The prevalence was higher in men, with wasting, stunting, and underweight at 14.2, 4.1, and 27.6%, respectively. Unprotected drinking water was associated with stunting [odds ratio = 0.68; 95CI (0.50, 0.92)]. Being male is another factor (aOR = 2.04, 95% CI: 1.13, 3.68). Lack of prenatal care follow-up was associated with a lower risk of wasting (OR = 2.20, 95% CI: 1.04, 4.64), while low birth weight (<2.5 kg), diarrhea, having a younger child, and being from a poor household were associated with wasting, stunting, and underweight. Other factors included body mass index (BMI) for age aOR = 2.11, 95% CI: (0.07, 0.895); maternal education: stunting [aOR = 1.52, 95% CI: (0.09, 0.89)], underweight [aOR = 1.97, 95% CI: (0.01, 0.73)], and open defecation, stunting [aOR = 1.62, 95% CI: (0.06, 0.32)], underweight [aOR = 1.92, 95% CI: (0.042, 0.257)]). Likelihood of being underweight increased with birth order (second born, aOR = 1.92, 95% CI 1.09–3.36; third born, aOR = 6.77, 95% CI 2.00–22.82).

**Conclusion:**

Inadequate dietary intake, illness, food insecurity, poor maternal and child care, poor hygiene and sanitation, and healthcare inaccessibility contribute to SAM.

## Introduction

Globally, over 500 million children under five are overweight or obese, while 45 million are wasted (too thin for their height) and 37 million are stunted (too short for their age). Undernutrition accounts for about half of all deaths in this age group. Concurrent wasting and stunting (WaSt) occur when a child experiences both conditions simultaneously (1). Currently, 50.5 million children under five are affected by wasting, including 17 million with severe wasting. In 2020, sub-Saharan Africa is estimated to have the highest prevalence of undernutrition, impacting 264.2 million people (24.1% of the population) (2). The likelihood of experiencing wasting, stunting, and underweight is influenced by insufficient breastfeeding practices, infectious diseases, lack of knowledge, poverty, family size, food insecurity, hygiene sanitation, poor maternal health, lack of antenatal care facilities, insufficient infrastructure, and poor absorption of nutrients (3, 4). Wasting is associated with energy deficiency, intake of carbohydrates and fats, breastfeeding practices, infectious diseases, lack of maternal nutrition knowledge, poor parenting, family size, food insecurity, hygiene sanitation, and low household income (4, 5). The major risk factors for stunting includes poor maternal health, lack of antenatal care facilities, insufficient feeding and care, and insufficient infrastructure and healthcare facilities (6, 7). A person may be underweight due to genetics, poor absorption of nutrients, increased metabolic rate or energy expenditure, lack of food (frequently due to poverty), low appetite, drugs that affect appetite, illness (physical or mental), or eating disorders such as anorexia nervosa (8, 9).

Stunting has more long-term or delayed developmental repercussions in comparison to wasting. Concurrent wasting and stunting (WaSt) is the term used to describe the state where wasting and stunting coexist (5, 10). The most severe forms of malnutrition occur when stunting, wasting, and underweight coexist (11). Compared to the risk of death associated with stunting and wasting independently, which were shown to be 1.47 and 2.30 times higher, respectively, than in healthy children, the risk of death in children with WaSt was found to be 12.75 times higher (12, 13).

According to a recent meta-analysis, the prevalence of WaSt varies from 0 to 8% across 84 countries, with fragile and conflict-affected areas showing the highest frequency (14). Wasting and stunting showed a significant correlation with the male sex, age of 12–23 months, infection, and having an underweight mother (15, 16). Both wasting and stunting have also been related to some risk factors, including low birth weight, low socioeconomic status, and maternal short height (17). Substantial research has also emphasized the risk factors and prevalence in low- and middle-income countries. Malnutrition has been a long-standing threat to children’s lives in developing countries (18). Areas that experience prolonged violence have a substantial influence on the frequency of malnutrition (19, 20). The primary causes of malnutrition—food instability and restricted access to healthcare—have been made worse by the disruption of essential services, especially food distribution and medical care (21–26). The most impacted are marginalized communities, which already have significant socioeconomic and health inequalities (27–29). As such, it is anticipated that, among children who are marginalized, malnutrition, especially wasting and stunting, will be prevalent. The number of impoverished children under the age of 5 in developing nations who are also impacted by circumstances that lead to underweight, wasting, and stunting who suffer from severe acute malnutrition in low-and middle-income countries remains unclear. The purpose of this systematic review was to assess the variables that simultaneously impact underweight, stunting, and wasting in children under five who are experiencing severe acute malnutrition in low- and middle-income countries.

## Methods

When conducting this investigation, the PRISMA (Preferred Reporting Items for Systematic Reviews) criteria were followed.

Among the databases that were searched were PubMed, MEDLINE, EMBASE, Cochrane Library, Web of Science, PsycINFO, CINAHL, Google Scholar, Scopus, MedRxiv pre-print, and the Science Direct search engine. A manual search was carried out in March 2022 via publisher and journal websites for research published from the inception of each database to February 28, 2023. The search plan was developed with input from a librarian. Medical Subject Headings (MeSH) terms were among the search terms used.

Study selection: This study comprises all studies involving children under five who have severe acute malnutrition.

Population: Children under five experiencing acute malnutrition.

Intervention: Nutritional supplementation programs (e.g., Ready-to-Use Therapeutic Foods).

Comparison: Standard care or no intervention.

Outcome: Improvement in height-for-age *z*-scores or reduction in prevalence of stunting.

### Inclusion and exclusion criteria

Children under five experiencing severe acute malnutrition were included in this review analysis. Exclusion criteria included reviewing secondary research, conference abstracts and posters, editorials/commentaries and protocols, unpublished literature, literature published before 2019, duplicated sources, irrelevant malnutrition questions/theses, dissertation manuscripts, case reports, and epidemiological studies. Underweight, stunting, and concomitant wasting in individuals over the age of five was also excluded.

### Study quality assessment

To determine the quality of the research that was a part of our systematic evaluation, we used the Newcastle–Ottawa Scale (NOS), which has a grading system ranging from 0 to 9. A quality score range of 0–3 was considered low, 4–6 was considered fair, and 7–9 was considered exceptional. The five evaluators—DAG, FCHT, CHGT, EMS, and TBF—rated the studies separately, and any differences were resolved by consensus.

### Extracting and analyzing data

Every reviewer extracted data using an identical data entry form. The original article was examined again to resolve differences among the five reviewers and reach a consensus. It was referred to as the consensus-building process.

#### Statistical analysis

The articles that were included in this systematic review for children under gender five all sought to look into different outcomes such as sex, being male sex, birthplace, child sexual abuse, parity, drug use, child labor, anemia, low birth weight, diarrhea, bottle feeding, initiation to complementary feeding, maternal age at birth and birth interval, family size, low income, marital status, poor diet, lack of animal protein, birth order, poor hygiene and sanitation, dietary diversity, vaccination status, sickness, illiteracy, food insecurity, water scarcity, maternal short stature, malaria, acute respiratory infection, divorce in the family, widowhood in the family, residing in a rural area, families’ meals/day. The results were documented by each original article’s predetermined objective and were assessed quantitatively.

#### Study characteristics

Table 1 summarizes the characteristics of the included studies in this systematic review. The study identified a total of 1,434,207 records from 12 studies (16, 19–21, 25, 26, 30–35) eligible for inclusion in the review. References (49–93) were excluded due to outdated publication years, incomplete results, and the geographic scope of the studies.

**Table 1:** Study characteristics.

Study population	Study type	Study design	Findings	Outcome	Determinants	Journals	Countries	Authors and publication year
Children under the age of five: (*n* = 2,399)	Original	Quant	Stunting, underweight, and wasting (13.5, 18.7, and 5.9%)	Concurrent wasting and stunting	Age of child, birthplace, child’s anemia, prematurity	BMC PH	Gambia	Asmare and Agmas (2022) (16)
Children under the age of five: (*n* = 209,60)	Original	Quant	Incomplete	Concurrent wasting and stunting	Diarrhea, bottle feeding, initiation to complementary feeding	Nurture	LMIC	Andrew et al. (2023) (13)
Children under the age of five: (*n* = 1,461)	Original	Quant	Stunting 42.5, 63.8%, wasted: 90% and wasted 58.6%	Concurrent wasting and stunting	Mother’s age at birth, education, and income level of parents	JMS	Yemen	Al-Sadeeq et al. (2024) (20)
Children under the age of five: (9854)	Survey	Quant	WaSt and under wt:10.9%, 15.4	Concurrent wasting and stunting	Birth interval, LBW, parent’s education	BMC Nut	Mozambique	Zaba et al. (2023) (21)
Children under the age of five: (33,650)	Original	Mixed	Grain/roots/tuber as bought food (OR = 9.487, CI = 1.182–76.138, *p* < 0.034)	Concurrent wasting and stunting	Diarrhea, large family size, low-income, marital status	MCN	Ethiopia	Sahiledengle et al. (2023) (25)
Children under the age of five (*n* = 566)	Original	Quant	Incomplete	Older age, low-income, illiteracy	Diarrhea, poor diet, lack of animal protein, large family	Elsevier	Ethiopia	Fufa (2022) (31)
PSC (*n* = 500)	Original	Quant	Stunting (40.6%).	Stunting	Dietary diversity, child age, family size, parent education	Elsevier	Ethiopia	Argaw et al. (2022) (33)
Children under the age of five: (*n* = 275)	Original	Quant	Wasting, stunting, and <wht: 11.1, 45.8, 25.5%	Undernutrition	Un vaccinated, sick child, lack of dietary diversity score	Hindi	Ethiopia	Sewnet et al. (26)
<5 (*n* = 5,355,000)	Global report	Survey	Stunting: 115 M, wasting: 52 M	Malnutrition	Poor feeding practices, poverty, decision over resources, illiteracy	City research	London	Hawakes and Fanzo (2017) (18)
Children under the age of five: (*n* = 352)	Original	Qual	Stunting, wasting, <wt. 45.795CI (9.9, 27.8) resp.	Undernutrition	Large family size, age of children, low income, illiteracy	JFNS	Ethiopia	Teklemariam et al. (2014) (23)
Children under the age of five: (*n* = 595)	Original	Quant	Maternal height aOR = 1.52, 95% CI 1.02–2.26, aOR = 2.37, 95% CI 1.29–4.35.	Underweight	Home delivery, pre-lacteal food, being male child, non-immunized	PHJ	Ethiopia	Rahel et al. (2017) (25)
Children under the age of five (*n* = 188)	Original	Quant	<wt with birth order (aOR = 1.92, 95% CI 1.09–3.36; aOR = 6.77, 95% CI 2.00–22.82).	Growth delay	Food Insecurity, Poor mother and child care, poor hygiene and sanitation	IOSR-JDMS	India	Sourajit et al. (2015) (48)
Children under the age of five (*n* = 450)	Original	Quant	Aged 6–23 m and 24–59 m stunted (aOR = 3.27, 95% CI 1.57 to 6.78 and aOR = 2.82, 95% CI 1.40–5.67)	Concurrent wasting and stunting	Illness, diarrhea, ARI, lack of vit. Suppl, large family size, poor sanitation, illiteracy, male gender, diarrhea, low income, not breastfed	JNS	Yemen	Al-Taj et al. (2023) (19)
Children under the age of five (*n* = 1,091)	Original	Quant	St. (aOR = 1.71.95% CI 1.14–2.55) and <wt (aOR = 2.11, 95% CI 1.16–3.82) unproved toilet	Factors influencing undernutrition	Poor toilet facilities, Poor water, diarrhea, LBW, unvaccinated, large family, child age, non-exclusive BF	BMJ Glob Health	Australia	Hall et al. (2020) (35)
Children under the age of five (*n* = 9,270)	Original	Quant	Incomplete	Undernutrition	Diarrhea, low income, child’s age, being male, shortage of water	BMC	Rwanda, Uganda and Tanzania	Kingsley et al. (2019) (3)
Children under the age of five (*n* = 139,529)	Review	Quant	Incomplete results	Wasting and stunting	Maternal short stature, being male, and LBW	BMC	Niger	Kristin et al. (2021) (41)
Children under the age of five (*n* = 33,054)	Data analysis	Multilevel	Incomplete results	Concurrent wasting and stunting	Malaria, diarrhea, ARI, being male, child’s age	MCN	Uganda	Gloria et al. (2021) (38)
Children under the age of five (*n* = 450)	Data analysis	Mixed level	Incomplete finding	Concurrent wasting and stunting	Being male, not breastfed, diarrhea, low income.	JNS	Yemen	Mansour et al. (2023) (16)
Children under the age of five (*n* = 5,753)	Original	Quant	Incomplete	Concurrent wasting and stunting	Higher birth order, income, low education levels of caregiver	IJPH	Ethiopia	Tamir et al. (2022) (42)
Children under the age of five (*n* = 240)	NFH Survey	Multilevel	Incomplete	Stunting and wasting	Income, Family size, birth interval, child age, diarrhea, unvaccinated, ECF	JN	Ghana	Francis et al. (2023) (43)
Children under the age of five (*n* = 4,764)	Original	Quant	Incomplete	Stunting	Divorce of widowship in the family, gender, rural residence, income, illiteracy, maternal age	JRHS	Indonesia	Hastin et al. (2021) (47)
Children under the age of five (*n* = 9,218)	Data review	Multilevel	Cont. WatSt. 24.8%, St. Wat. and <Wt.38,9.4, 25.2%	Multiple nut deficiencies	Birth order, sex, age of child, parity, hygiene and sanitation	BMC	Ethiopia	Geda et al. (2021) (32)
Children under the age of five (*n* = 9,218)	Original	Quant	Stunting 38.4%	Stunting	Being male, age, family’s meals/day	BMC	Indonesia	Ramli et al. (2009) (44)
Children under the age of five (*n* = 8,064)	Review	Quant	Incomplete	Stunting and wasting	ARI, M.BMI, income, sex, age, LBW, poverty, illiteracy	PLoS One	Indonesia	Hayani et al. (2023) (45)
Children under the age of five (*n* = 1,260)	Original	Quant	HH FI, (OR = 2.079, CI = 1.182–3.658)	Stunting	Birth place, BFP, LBW, child sex, illiteracy	CCHD	China	Jiang et al. (2014) (46)
Children under the age of five (*n* = 400)	Original	cross-sectional	Stunting:24.7%, Pre-mensuration mum (OR = 5.015, CI = 1.257–20.011, *p* < 0.022)	Factors influencing undernutrition	Poverty, failure to breastfeed exclusively, poor nutrition during pregnancy	Nutrient	Nepal	Giri et al. (2023) (34)

Across all studies, a total of 1,434,207 children under five were enrolled, with a mean age of 3.50 years and 57% being male. All included studies were conducted in low- and middle-income countries. The remaining studies were conducted in Europe, the United States, and Canada. All the included studies were original and quantitative and were published after 2019. All the studies included in this review used mid-upper arm circumference, weight-for-height, height-for-age, and weight-for-age measurements. The quality of the studies reviewed ranged from moderate to high, with each study achieving a score greater than 6 out of 9 on the no-quality assessment.

#### Data collection process

Data from reports was gathered by manual search and direct data extraction from journal articles and other study reports. This entails the use of structured data-gathering forms, which can be created using bespoke data systems, article forms, or electronic forms. Five reviewers independently collected data from each report, and after reading the reviewed manuscript, all the authors gave their approval.

##### Study risk of bias assessment

To determine the possibility of bias in the study, the five reviewers each worked on or evaluated the study on their own. The critical evaluation of the included articles was finished by those five reviewers (DAG, FCHT, CHGT, ESM, and TBF), and one reviewer (AG) dealt with conflicts. All reviewers used a set of 12 randomly selected articles to cross-check the reviewer’s conclusions to ensure quality control.

##### Synthesis methods

**Figure 1:**
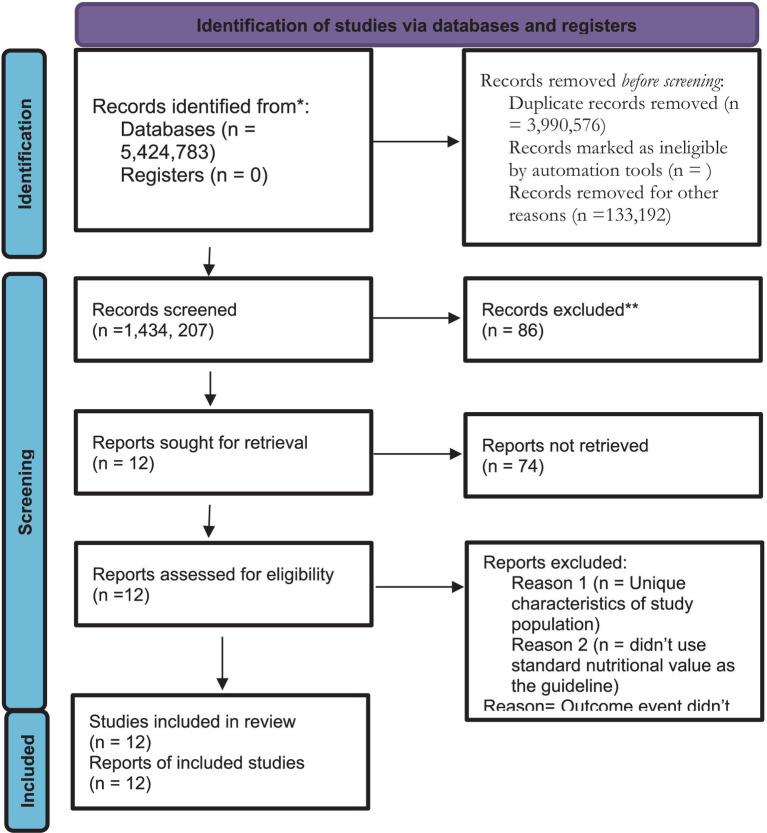
PRISMA flow chart diagram 2020.

The following method was used to synthesize the study’s eligibility: Figure 1 shows the PRISMA flow diagram for systematic reviews, which includes database searches and screening. Additional records were found by hand searching through Science Direct, Google Scholar, and journal websites, as well as through organizations like the World Health Organization. The study complied with Cochrane guidelines for fast reviews and World Health Organization guidelines for treating malnutrition. The stages of the review included knowledge synthesis, report dissemination, procedure construction, literature search, research selection and screening, data extraction, risk of bias evaluation, and question refinement. The results of individual investigations and syntheses were tabulated or graphically displayed using quantitative data synthesis such as cross-sectional and analytical design. We summarized the findings in this systematic review and provided expert advice to support the decision(s). This systematic review did not, however, address the model(s), method(s) for identifying the presence and level of statistical heterogeneity, or software package(s) that were used. It did not employ any techniques to investigate the reasons behind the heterogeneity among subgroup studies, which would have entailed dividing all participant data into smaller groups frequently to compare them. However, a sensitivity assessment was performed using the analytical approach, assumptions from earlier research outcomes, and predictive variables from independent investigations.

##### Certainty assessment

We evaluated study limitations, heterogeneity, inconsistency, indirectness, imprecision, and publication bias in order to determine the degree of certainty in the body of evidence supporting a conclusion.

### Technical terms

The PICO is a framework used to formulate research questions, especially in health and clinical settings. It stands for:

- Population
- Intervention
- Comparison
- Outcome

Intervention: Nutritional supplementation programs (e.g., ready-to-use therapeutic foods).

Comparison: Standard care or no intervention.

Outcome: Improvement in weight-for-height *z*-scores or recovery rates from acute malnutrition.

When defining PICO for wasting, stunting, and underweight among children under five, here’s how it might look:

1. Wasting:

Population: Children under 5 years old experiencing acute malnutrition.

Intervention: Nutritional supplementation programs (e.g., ready-to-use therapeutic foods).

Comparison: Standard care or no intervention.

Outcome: Improvement in weight-for-height *z*-scores or recovery rates from acute malnutrition.

2. Stunting:

Population: Children under 5 years old at risk of chronic malnutrition.

Intervention: Growth monitoring and nutrition education programs.

Comparison: Usual care or no intervention.

Outcome: Improvement in height-for-age *z*-scores or reduction in the prevalence of stunting.

3. Underweight:

Population: Children under five classified as underweight (weight-for-age *z*-scores).

Intervention: Integrated management of childhood illness (IMCI) with nutritional support.

Comparison: Routine healthcare without specific nutritional interventions.

Outcome: Change in weight-for-age *z*-scores or reduction in the prevalence of underweight.

This structure helps in designing studies, evaluating interventions, and analyzing outcomes related to child malnutrition effectively.

## Results

In their pooled sample, the prevalence of wasting was 0.66 (95% CI: 0.64, 0.67) for children under 2 years old and 14% (95% CI: 13, 14) for children aged 2–4. In 87 nations, prevalence ratios were less than one, suggesting a reduced prevalence in children over two. Additionally, in 68 countries, the prevalence was statistically significantly lower than one at a non-adjusted 5% level. For boys and girls, as well as the wealthiest and poorest households, the prevalence of wasting was generally lower in children under two (16). Out of the 2,399 children under five that were examined in this study, 13.5, 18.7, and 5.9% experienced underweight, wasting, and stunting, respectively. The majority of the children (40.1%) came from the Gambia’s Brikama local government region; male children made up 52.9% of the child population, and 63.3% of the children lived in urban areas. In light of the other predictors, the odds ratios (OR) for the relationships between stunting and underweight, underweight and wasting, and stunting and wasting were 15.87, 46.34, and 1.75, respectively. Compared to children who had a small birth size, the calculated odds ratios for stunting, underweight, and wasting outcomes were 0.965, 0.885, and 0.989 times higher for children with an average birth size (17). The study findings provide evidence for the co-existence of stunting among severely wasted children early in life. Wasting and stunting both need to be addressed simultaneously to reduce associated short- and long-term irreversible consequences (21). Compared to female and urban-born children, male children and those born in rural areas were more likely to suffer from severe and mild stunting, wasting, and underweight. The percentage of stunted, wasted, and underweight male children was 27.6, 4.10, and 14.2%, respectively. Stunting was linked to unprotected drinking water [odds ratio (OR) = 0.68; 95% confidence interval (CI): 0.50, 0.92].

A higher risk of underweight was linked to having a mother who was under 20 years old at birth (OR = 0.66; 95% CI: 0.45, 0.97) and being a male child (OR = 2.04; 95% CI: 1.13, 3.68). The lack of prenatal care follow-up (OR = 2.20; 95% CI: 1.04, 4.64) was linked to wasting, whereas diarrhea, low birth weight (<2.5 kg), a child’s younger age, and three or more under-five children were substantially linked to underweight, wasting, and stunting in a household. Increased rates of stunting, wasting, and underweight were linked to several variables, including being a male child, being born in a rural region, having unprotected drinking water, being smaller at birth, not receiving prenatal checkups, having diarrhea, and having low household affluence. Therefore, policymakers needed to implement initiatives that prioritize utilizing prenatal care services, increasing household wealth, and enhancing access to safe drinking water to reduce stunting, wasting, and underweight more quickly (30). The single composite index of anthropometric indicators showed that 49.0% (19.8% moderately and 29.2% severely) of sampled children were undernourished. In the Brant test of proportional odds model, the null hypothesis that the model parameters equal across categories was rejected. Compared to ordinal regression models, partial proportional odds model showed an improved fit. A child whose mother’s body mass index is less than 18.5 kg, is from a poor family, whose father is without education, and who is male has a severe under-nutrition status that is 1.4, 1.8, 1.2, and 1.2 times, respectively, more likely to be worse than the reference group.

The authors conclude that the fitted partial proportional odds model indicated that age and sex of the child, maternal education, region, source of drinking water, number of children under five, mother’s body mass index and wealth index, anemic status of child and, fever in the child 2 months before the survey had a negative effect (36). Child’s age [confidence intervals for (wasting = 0.02, 0.007; stunting = 0.042, −0.011)] and sex [confidence intervals for (underweight = 0.530, −0.151; stunting = 0.936, −0.243) (underweight = −0.025, 0.002)], maternal measuring mid upper arm circumference (MUAC) [confidence intervals for (wasting = 0.189, 0.985; BMI-for-age = 0.077, 0.895), maternal education (stunting, 0.095, 0.897; underweight = 0.120, 0.729), and open defecation (stunting = 0.055, 0.332; underweight = 0.042, 0.257)] were discovered to be significantly linked to anthropometric indicators. In contrast to some research, the anthropometric parameters of the aforementioned child do not indicate that maternal dietary diversity is significant (37). The findings from our study indicated that having diarrhea, having an acute respiratory infection (ARI), not having water availability all year, and not attending monthly child growth monitoring sessions were associated with undernutrition among children aged 0–59 months. Interventions aimed at improving undernutrition in these disadvantaged communities should target all children, especially those children from households with poor sanitation practices (38). In children under five, the coexistence of stunting with overweight/obesity ranged from 0.8% in the United States to over 10% in Ukraine and Syria, while the prevalence of coexisting wasting with stunting ranged from 0.1% in most of the South American countries to 9.2% in Niger. A decrease in the prevalence of coexisting forms of malnutrition (CFM) was observed in all countries except Indonesia. Studies in China and Indonesia showed a positive association between rural area and city of residence and coexistence of stunting with overweight/obesity. Evidence for other risk and protective factors for the CFM is too minimal or conflicting to be conclusive (3).

## Discussion

This systematic review examined the factors that contribute to concurrent stunting, underweight, and wasting in children under five who are suffering from severe acute malnutrition in low- and middle-income countries. Concurrent wasting and stunting were defined as the proportion of measurements at a specific age when a child was both wasted and stunted at the same time (13). This combination represents a severe form of malnutrition among children, especially in vulnerable groups impacted by conflict, as this systematic review has shown (11, 13, 17, 21, 22, 39). As our recent fast analysis demonstrated, the proportion of concurrent wasting and stunting varies by country. For example, in Gambia, 13.5, 18.7, and 5.9% of children suffered from stunting, being underweight, and wasting, influenced by age of the child, child’s anemia, place of birth, and small birth size (WaSt) (11); in low- and middle-income countries, the prevalence of wasting, stunting, and underweight were 10.0, 29.2, and 13%, respectively, while diarrhea, bottle feeding, and initiation to complementary feeding are related to wasting and concurrent stunting (13). In Yemen, the prevalence of severe stunting, moderate stunting, and severe wasting was 42.5, 63.8, and 58.6%, respectively, with 10.7% experiencing concurrent wasting and stunting. Concurrent wasting and stunting were associated with mother’s birth age, birth interval, parents’ education level, and socioeconomic situation (17). In Mozambique, a single study found that the prevalence of concurrent wasting, stunting, and underweight was 10.9, 15.4, and 60.9%, respectively. Concurrent wasting and stunting were associated with birth intervals <2 years, mothers’ education, and low birth weight (17). In Ethiopia, the prevalence of stunting, underweight, wasting, and anemia was 38, 25.2, 9.4, and 58%, respectively (39). Another study in Ethiopia found that the prevalence of simultaneous wasting, stunting, and underweight was 11.1, 45.8, and 25.5% (22).

Ethiopia also has a high frequency of concomitant WaSt (5.8%), wasting (16.8%), stunting (53.9%), and underweight (36.9%) (40). Another multidimensional nutrition deficiency assessment conducted in Ethiopia found that the prevalence of stunting, underweight, and wasting was 38, 25.2, and 9.4%, respectively. Fifty-eight percent of children were anemic, and the prevalence of concurrent stunting and anemia was 24.8% (36), whereas birth order, parity, age and sex of child, parental education, religion, household wealth index, type of family structure, hygiene and sanitation, child feeding practice, and health service utilization, were the key variables, while poor nutrition, diarrhea, big family size, age of breastfeeding, lack of animal protein, illness, non-vaccination, and an inadequate dietary variety score influenced concurrent WaSt (36, 39).

In Niger, 14% of children were wasted, 80% were stunted, and 12% were simultaneously wasted and stunted (41). In India, the prevalence of WaSt has dropped from 8.7% in 2005–2006 to 5.2% in 2019–2020. From 6 to 18 months, the proportion of concurrent WaSt children increased rapidly, peaking at 19 months with 8% (42). In Ghana, the prevalence of stunting and wasting in children under five was 12.5 and 27.5%, respectively (43). In Indonesia, the prevalence of stunting and severe stunting was 29 and 14.1%, respectively. Stunting was seen in 38.4% of children aged 0–23 months (44). Factors linked with stunting included child age, sex, number of family meals/day, maternal BMI, birth weight, maternal weight and height, mother’s education/illiteracy, and poor household status (44, 45). Finally, in China, the prevalence of stunting and severe stunting was 27.0 and 13.2%, respectively (34, 46), and the place of residence, caregiver’s education, child’s gender, low birth weight, and duration of exclusive breastfeeding were risk factors for stunting (35). Even though there is a widespread list of factors influencing concomitant wasting, stunting, and underweight, there is a dearth of sequential research on the coexisting factors influencing underweight, stunting, and wasting in children under five who suffer from severe acute malnutrition in low- and middle-income countries. In this systematic review, concurrent WaSt was found to more likely affect boys than girls, although the extent of this disparity varies and is more prominent in certain circumstances (3, 16, 17, 25, 36, 38, 39, 41, 44, 45, 47). This is primarily because boys typically consume more calories and have larger body frames and sizes than girls, and a larger body mass requires more food. Therefore, they might not have the same tolerance during a food crisis as girls do. These results indicate that malnutrition is still a major public health problem among children under five. The three main factors that are commonly associated with stunting, wasting, and underweight are the child’s age, anemia level, and birth type. For children under five, being undersized at birth was strongly linked to an increased risk of stunting, underweight, and wasting. Additionally, wasting, WaSt, and underweight were associated with cough. Moreover, wasting was substantially correlated with maternal age, occupation, and being a child from a poor family.

### Conclusion

Inadequate dietary intake, poor-quality diet, infectious disease, poor water quality, child’s age, anemia level, birth order/type, large family, prematurity, a child from a malnourished mother, being a boy, hygiene and sanitation poverty, food insecurity, climate change, drought and conflict, healthcare inaccessibility, and poor cultural feeding practices are the factors that are usually associated with stunting, wasting, and underweight.

### Recommendation

Addressing these factors holistically with interventions across health, education, and economic sectors can improve children’s growth and health outcomes.

### Strengths and weaknesses of the study

#### The strengths of the study

The strengths of this systematic review lie in its comprehensive approach, reproducibility, and precise articulation of outcomes. A concise background explanation outlining the reasoning behind the review, literature search, and reference management are given below:

- Both the review question and the topic sentences were stated clearly.
- A smart title (specific, quantifiable, attainable, reasonable, and time-bounded) could provide access to the review method.

#### Weakness of the study

The exclusion of certain features of the research population or unique characteristics of the study population and literature from earlier years of publication, incomplete findings, the lack of standard nutritional values as a guide in some of the analyzed papers, and inconsistent outcome events are some of the weaknesses.

#### Registration and protocol

Although there was no registration for this systematic review, eligibility was checked via screening.

### Limitation

There are some limitations in this review. First, the shortcoming of this systematic review was that its search was not exhaustive. Second, only one reviewer was used at a time. Third, the possible appraisal and selection procedure was without blinding. Fourth, interpretation of the data may have been constrained or cautious and, lastly, there is no precise definition on what defines a systematic review.

## Data Availability

The raw data supporting the conclusions of this article will be made available by the authors, without undue reservation.
